# Supplementary material for: Preparation, Characterization and In Vitro Stability of a Novel ACE-Inhibitory Peptide from Soybean Protein
Source: Foods. 2022 Sep 1;11(17):2667. doi: 10.3390/foods11172667 (PMC9455805; doi:10.3390/foods11172667)
Supplement: Supplementary file 1 [file foods-11-02667-s001.zip › foods-1852536-supplementary.pdf]

foods

Supplementary Materials

Preparation, Characterization and In Vitro Stability of a Novel ACE-Inhibitory Peptide from Soybean Protein

Sara Sangiorgio [1], Nikolina Vidović [1], Giovanna Boschin [2], Gilda Aiello [2,3], Patrizia Arcidiaco [4], Anna Arnoldi [2], Carlo F. Morelli [1], Marco Rabuffetti [1], Teresa Recca [4], Letizia Scarabattoli [1], Daniela Ubiali [5] and Giovanna Speranza [1,*]

[1] Department of Chemistry, University of Milan, via Golgi 19, 20133 Milan, Italy
[2] Department of Pharmaceutical Sciences, University of Milan, via L. Mangiagalli 25, 20133 Milan, Italy
[3] Department of Human Science and Quality of Life Promotion, Telematic University San Raffaele, 00166 Rome, Italy
[4] Centro Grandi Strumenti, University of Pavia, via Bassi 21, 27100 Pavia, Italy
[5] Department of Drug Sciences, University of Pavia, Viale Taramelli 12, 27100 Pavia, Italy
* Correspondence: giovanna.speranza@unimi.it; Tel.: (+39)-02503-14097

Supplementary Materials Summary

1. Degree of Hydrolysis (DH) 2
1.1. Samples Preparation and Analysis 2
1.3. Amino Acids profile of SPI and Nitrogen Content of the Peptide Bonds (N_{pb}) Calculation. 3
1.4. Samples Analysis and Amino Nitrogen (AN) Calculation 4
2. Foaming Properties 4
3. ACE-Inhibitory Activity Plots 5
4. RP-HPLC 7
5. NMR Analysis: ^{1}H and TOCSY Spectra of A1 Fraction 7
6. Amino Acid Analysis 8
7. Edman Degradation Analysis 9
8. Peptides Loading on Resin Support for Solid Phase Synthesis 14
9. Spectroscopic Characterization of Synthesized and In Vitro Digested Peptides 14
9.1. ALKPDNR ESI-MS (Positive Detection) 14
9.2. VVPD ESI-MS (Positive Detection) 15
9.3. NDRP ESI-MS (Positive Detection) 16
9.4. NDTP ESI-MS (Positive Detection) 17
9.5. In Vitro Digested NDRP ESI-MS (Positive Detection) 18
9.6. ^{1}H NMR of NDRP 19
9.7. ^{13}C NMR of NDRP 20
9.8. NDRP HR-MS (Positive Detection) 21

1. Degree of Hydrolysis (DH)

1.1. Samples Preparation and Analysis

Degree of hydrolysis (DH) of the obtained SPH (A, F, A+F) was determined using the trinitrobenzenesulfonic acid (TNBS) method. Briefly, an aliquot (250 µL) of the examined sample solution prepared in SDS (1% *w*/*v*) was diluted in 0.21 M sodium phosphate buffer (pH 8.2) (2 mL) and TNBS aqueous solution (0.1% *w*/*v*) (2 mL) was added afterward. Tightly secured tubes containing samples were mixed and incubated in the dark, at 50 °C for 60 min. The reaction was stopped by the addition of 0.1 M HCl (4 mL). Samples were allowed to cool at room temperature for 30 minutes and then submitted to spectrophotometric analysis of absorbance at 340 nm, using a JASCO V-630 UV-VIS Spectrophotometer (JASCO Europe, Cremella, Italy). Each assay was performed in triplicate. L-Leucine (0-2 mM) was used to generate a standard curve.

1.2. Standard Curve Generation

L-Leucine (0-2 mM) was used to generate a standard curve.

Table S1. L-Leu samples analysis for calibration curve.

[L-Leu] (*mM*)		A (*AU*)		Average A (*AU*)	Std. Dev. (*AU*)
0	0.0763	0.0533	0.0448	0.0582	0.0163
0.5	0.112	0.113	0.102	0.109	0.00601
1	0.170	0.177	0.170	0.173	0.00433
1.5	0.251	0.260	0.259	0.257	0.00481
2	0.317	0.317	0.319	0.318	0.00124

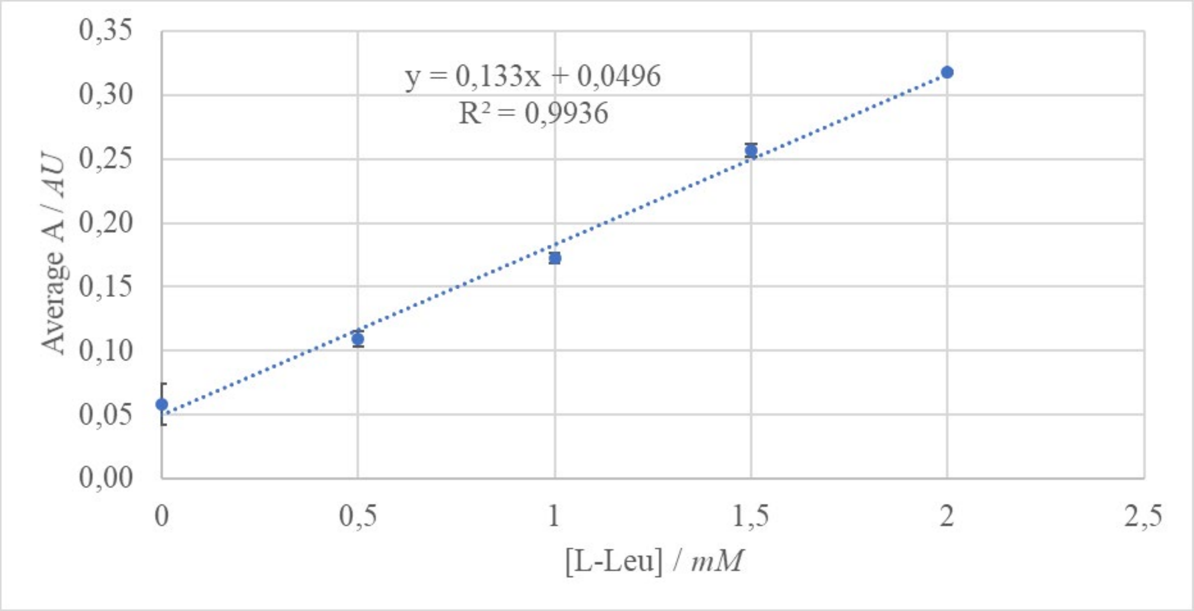

Figure S1. L-Leu calibration curve.

Moreover, the molecular mass of nitrogen (14.007 g mol^{-1}) was multiplied by each concentration value of L-Leu. Thus, the absorbance values were related to the amino nitrogen content (AN) of each L-Leu sample, generating a second calibration curve.

Table S2. $AN_{L\text{-}Leu}$ calibration curve.

$AN_{L\text{-}Leu}$ (*mg L⁻¹*)	Average A (*AU*)	Std. Dev. (*AU*)
0	0.0582	0.0163
7.0035	0.109	0.00601
14.007	0.172	0.00433
21.0105	0.257	0.00481
28.014	0.318	0.00124

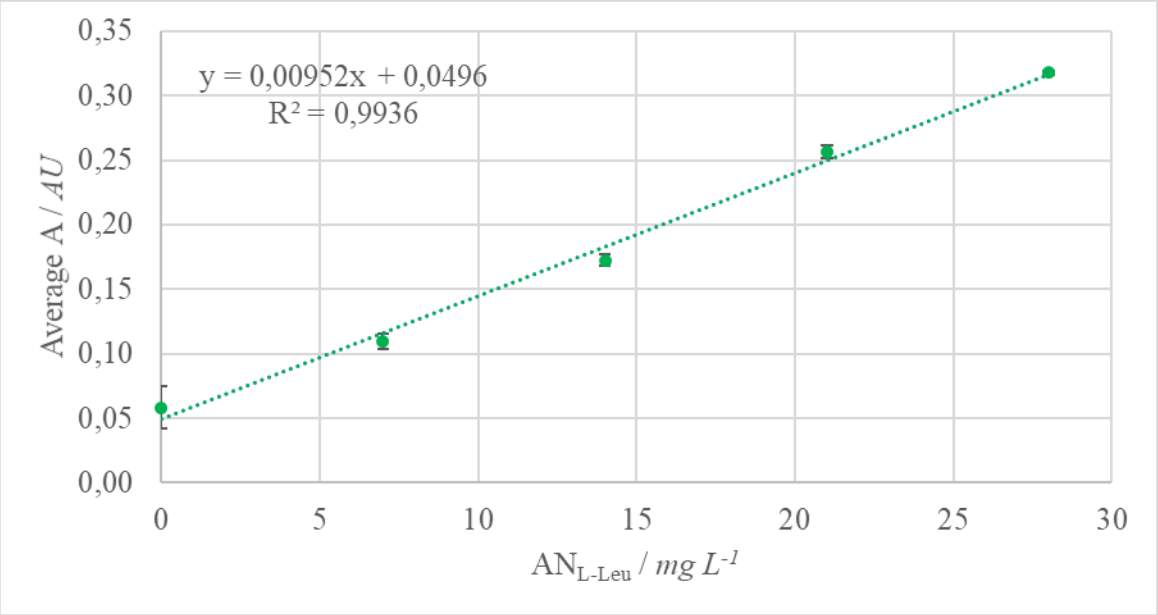

Figure S2. $AN_{L\text{-}Leu}$ calibration curve.

1.3. Amino Acids Profile of SPI and Nitrogen Content of the Peptide Bonds (N_{pb}) Calculation

The nitrogen content of the peptide bonds (N_{pb}) in the protein substrate (SPI) was calculated by assuming that all the nitrogen content in the starting material was related to proteins and by multiplying the molecular mass of nitrogen (14.007 g mol^{-1}) for the moles sum of each amino acid (AA) present in the SPI, as reported in the amino acids profile provided by the supplier.

Table S3. Amino acids profile of SPI and N_{pb} calculation.

AA	m (*g 100g⁻¹*)	MW (*g mol⁻¹*)	mol (*mol 100g⁻¹*)	mol sum (*mol 100g⁻¹*)	N_{pb} (*mg g⁻¹*)
Asp	9.49	133	0.071	0.63	88.01
Thr	3.11	119	0.026		
Ser	4.27	105	0.041		
Glu	15.19	146	0.10		
Gly	3.31	75	0.044		
Ala	3.8	89	0.043		
Val	4.45	117	0.038		
Met	1.3	149	0.009		
Ile	3.48	131	0.027		
Leu	6.43	131	0.049		
Tyr	3.31	181	0.018		
Phe	4.33	165	0.026		
Lys	5.2	146	0.036		
His	2.06	155	0.013		

Arg	6.18	174	0.036
Pro	3.99	115	0.035
Trp	1.25	204	0.0061
Cys	0.86	121	0.0071

1.4. Samples Analysis and Amino Nitrogen (AN) Calculation

Table S4. Starting isolate soy proteins (SPI) and hydrolysates samples (A; F; A+F) analysis.

Sample	A (*AU*)			Average A (*AU*)	Dev. Std. (*AU*)
SPI	0.0701	0.0620	0.0652	0.0658	0.00407
A	0.165	0.180	0.174	0.173	0.00716
F	0.283	0.278	0.277	0.279	0.00324
F+A	0.261	0.265	0.265	0.264	0.00237

The slope and the intercept of the $AN_{L\text{-}Leu}$ calibration curve equation are used calculate both the amino nitrogen of the protein substrate before hydrolysis (AN_1) and the amino nitrogen content of the protein substrate after hydrolysis (AN_2), which must be expressed as mg g^{-1} of proteins.

Table S5. AN calculation.

Sample	AN (*mg* L^{-1})	AN error (*mg* L^{-1})	[Conc.] (*mg* mL^{-1})	AN (*mg* g^{-1})	AN error (*mg* g^{-1})
SPI	1.70	1.31	0.74	2.30	1.77
A	13.0	2.16	0.74	17.5	2.92
F	24.1	2.26	0.74	32.6	3.05
F+A	22.5	2.10	0.66	34.1	3.17

1.5. Degree of Hydrolysis (DH) Calculation

DH values were calculated using the following equation:

$$\text{DH (\%)} = 100 \left(\frac{\text{AN}_2\text{-AN}_1}{\text{N}_{\text{pb}}} \right) \quad (1)$$

Table S6. DH calculation.

Sample	DH (%)	DH error (%)
A	17	5
F	34	5
F+A	36	6

2. Foaming Properties

Foaming capacity (FC) was determined as the measure of the foam volume (FV) generated by SPH aqueous solutions (10% *w*/*v*) present in a 100 mL graduated cylinder after flushing with a constant and homogeneous nitrogen flow (1.5 L min^{-1}, 5 minutes). To check the foam stability (FS), foam volumes were recorded at 1-, 5-, 10- and 30-minutes intervals (FV_t). FC and FS were calculated according to the following equations:

$$FC = (FV / FV_R) \times 100; \qquad FS = (FV_t / FV_0) \times 100. \quad (2)$$

where FV_R represents to the foam volume generated by a reference human serum albumin aqueous solution (2% *w*/*v*) submitted to the same conditions and FV_0 is the starting foam volume.

Table S7. Foaming capacity (FC) and foaming stability (FS) over time.

	FC (%)	1 min FS (%)	5 min FS (%)	10 min FS (%)	30 min FS (%)
**A**	91	15	7	-	-
**F**	73	36	16	15	9
**A+F**	53	13	-	-	-

3. ACE-Inhibitory Activity Plots

ACE-inhibitory activity plots were constructed by evaluating hippuric acid formation from hippuryl-histidyl-leucine, a mimic substrate for angiotensin I, by HPLC analysis at different concentrations of inhibitor (0, 86.3, 172.5, 345.1, 690.1, 1035.2 μg mL^{-1}).

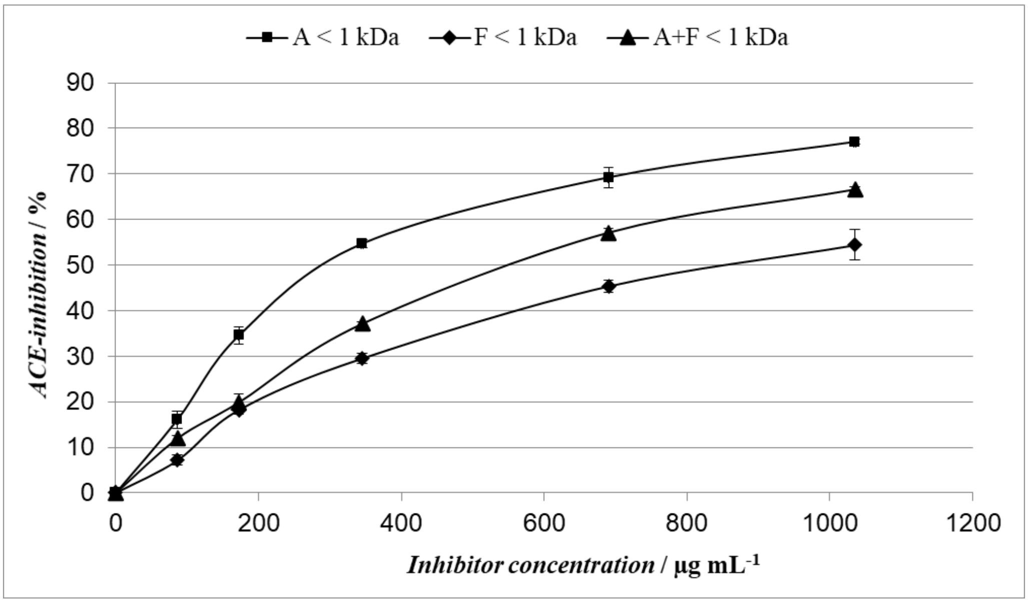

Figure S3. ACE-inhibitory activity curves comparison of ultrafiltered hydrolysates A < 1 kDa, F < 1 kDa and A+F < 1 kDa. .

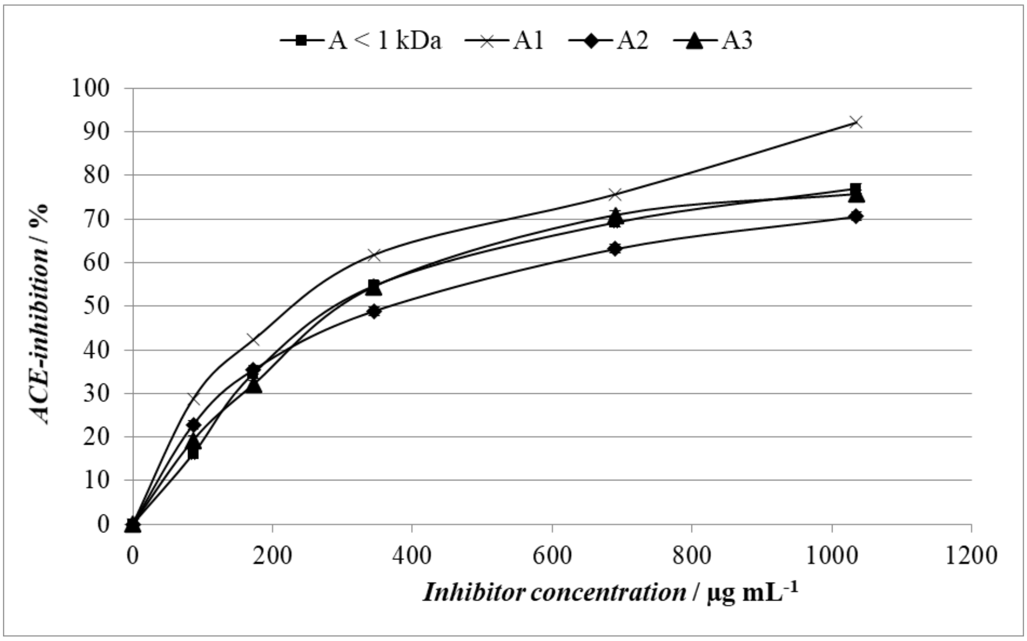

Figure S4. ACE-inhibitory activity curves comparison of ultrafiltered hydrolysates A < 1 kDa and fractions purified via semipreparative RP-HPLC from A < 1 kDa (A1, A2 and A3).

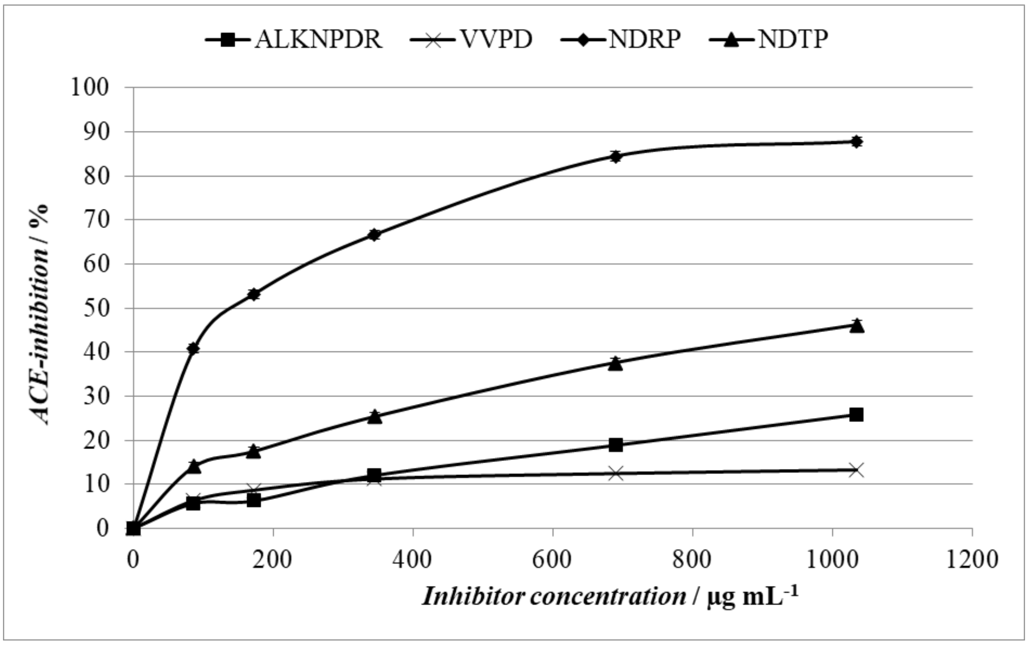

Figure S5. ACE-inhibitory activity curves comparison of synthesized peptides (ALKPDNR, VVPD, NDRP and NDTP).

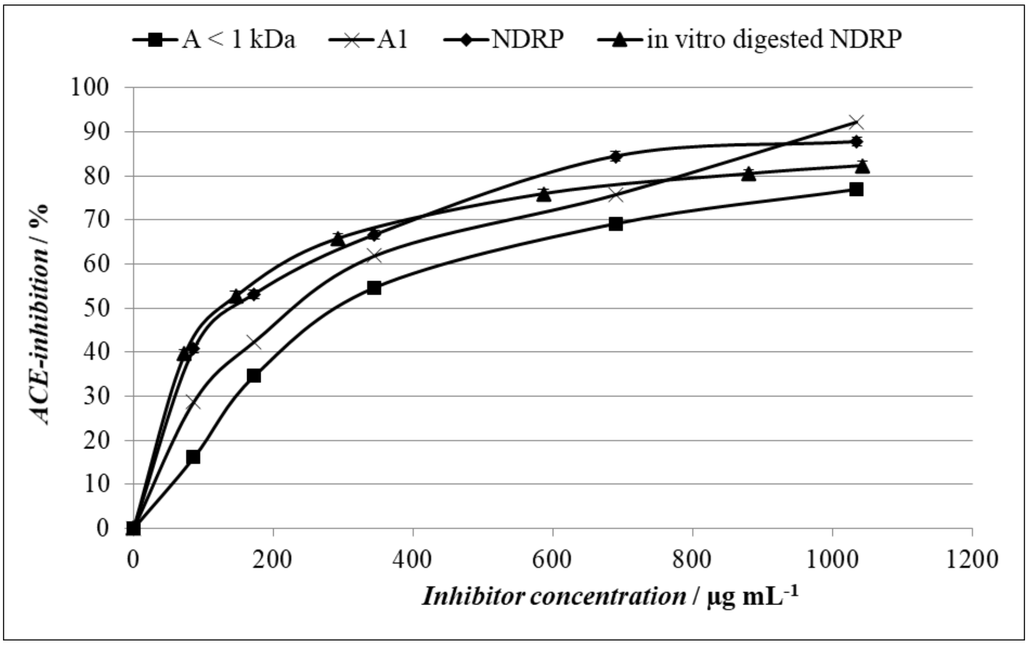

Figure S6. ACE-inhibitory activity curves comparison of ultrafiltered hydrolysates A < 1 kDa, purified fraction A1, synthesized peptide NDRP and in vitro digested NDRP.

4. RP-HPLC

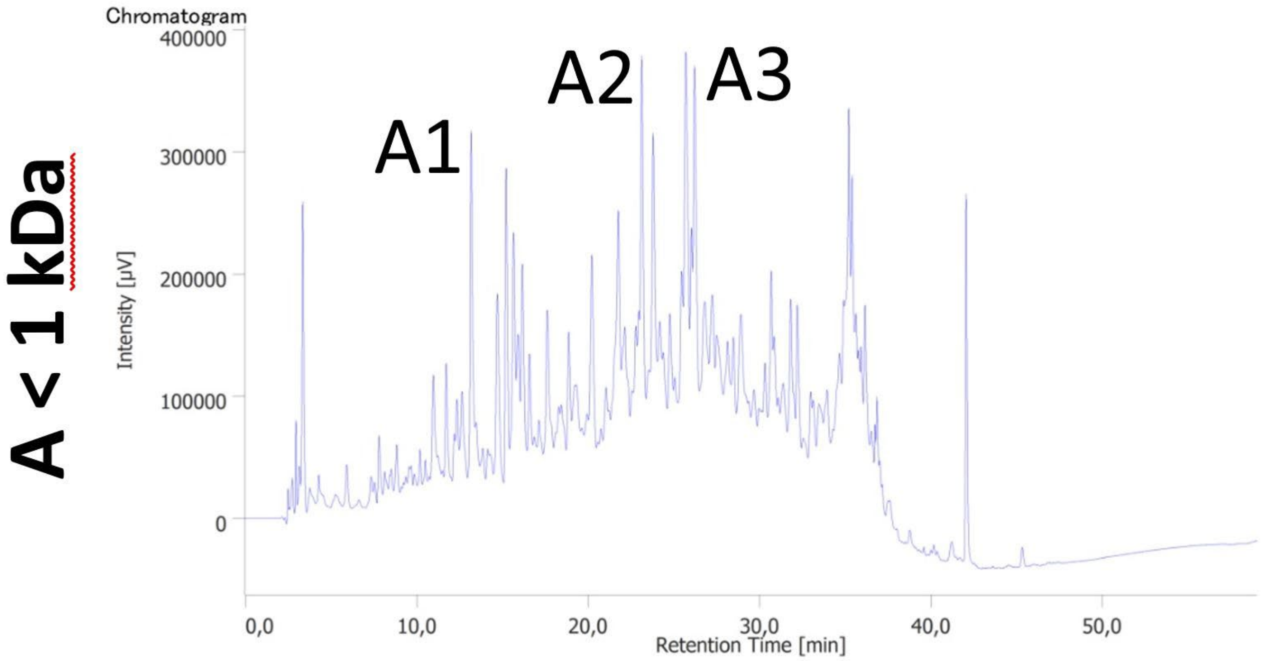

Figure S7. RP-HPLC chromatogram on reverse phase Jupiter 10 µm Proteo 90 Å column of active fraction A < 1 kDa.

5. NMR Analysis: ^{1}H and TOCSY Spectra of A1 Fraction

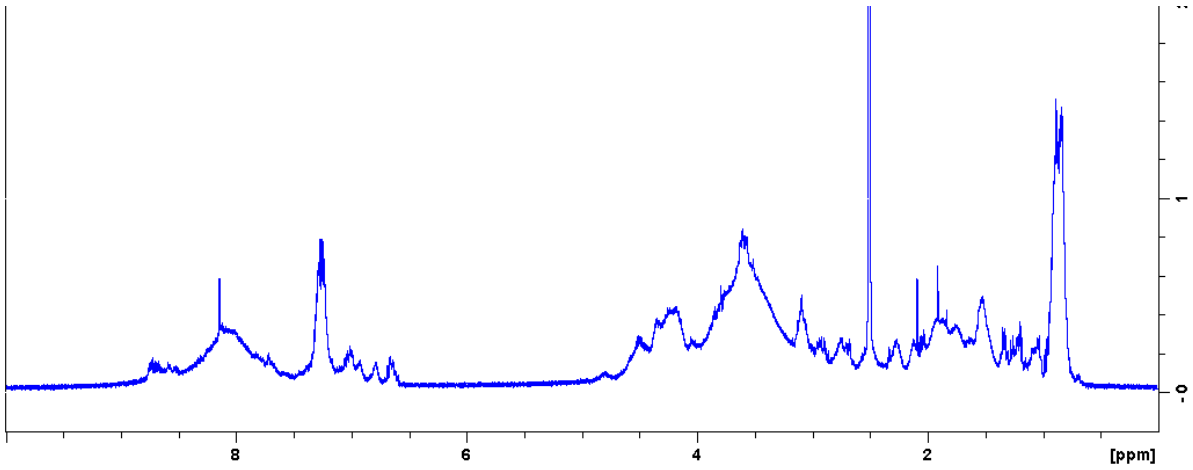

Figure S8. ^{1}H NMR (400 MHz; DMSO-d_6) spectrum of A1 sample.

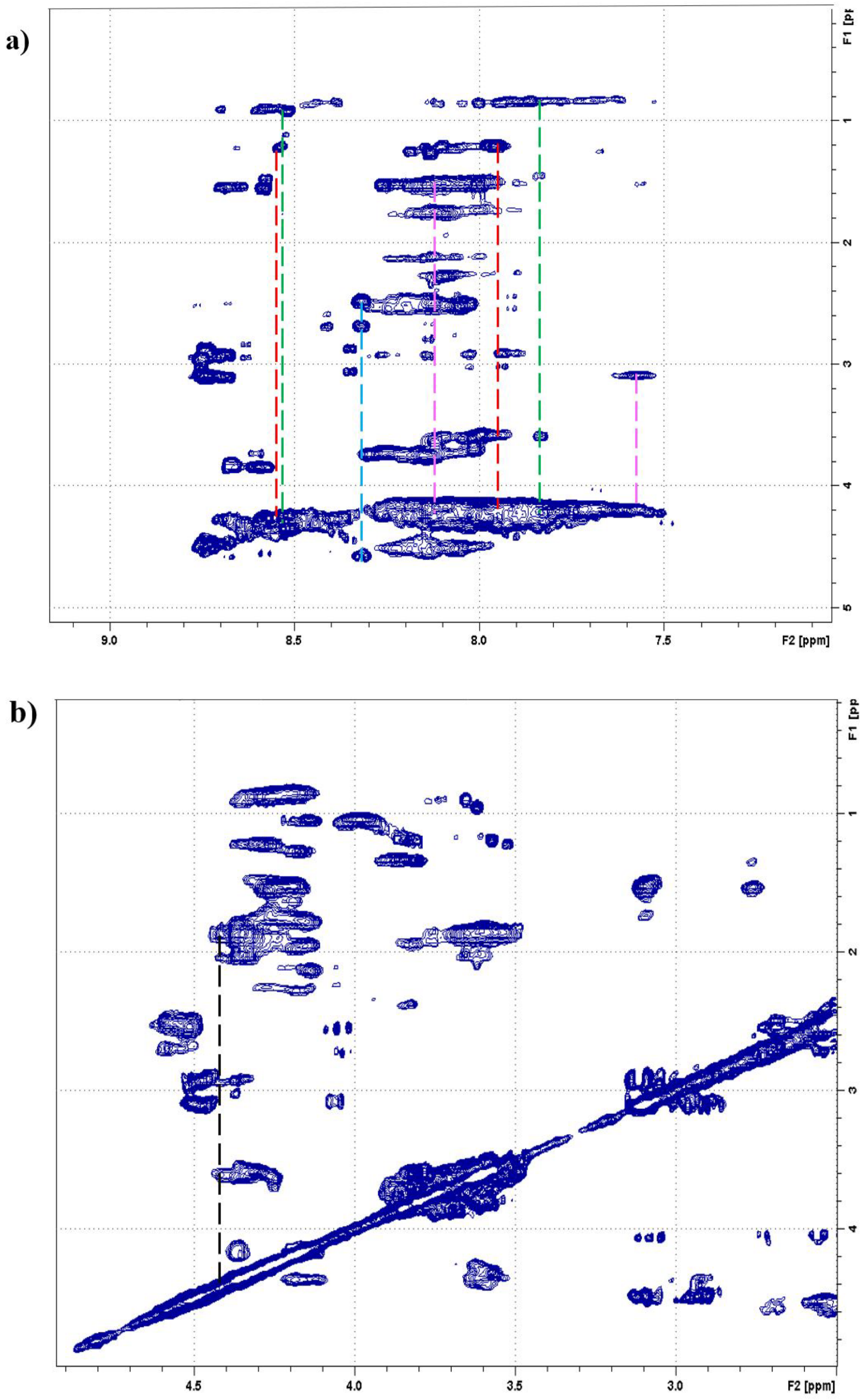

Figure S9. (**a**) NH-αH region and (**b**) αH-αH region of the TOCSY spectrum of the A1 sample; dashed lines correspond to the patterns of alanine (red), valine (green), aspartic acid (blue), arginine (pink) and proline (black).

6. Amino Acid Analysis

Fraction A1 (1.6717 mg) was hydrolysed in 6M HCl at 110 °C under nitrogen for 24 h in sealed tube. After hydrolysis, it was derivatized with orthophthalaldelyde (OPA) and 9-fluorenylmethyl chloroformate (Fmoc). OPA and Fmoc derivatives were analyzed by

Jasco X-LC Amino Acid Analyzer (Jasco Corporation, Tokyo, Japan) with a fluorescence detector (Jasco X-LCTM 3020FP) (excitation/emission at λ 340/446 nm and at λ 268/308 nm for OPA-amino acids and for Fmoc- amino acids, respectively). The amino acid analysis was performed in triplicate. Data were analyzed using chromatography software Chrom-NAV.

Table S8. Amino acids profile of A1.

Amino Acid (AA)	w_{AA} (*mg*)	w_{AA} / w_{tot} %
Asp	0.1646	8.482
Glu	0.3562	18.363
Ser	0.0926	4.775
Hys	0.0454	2.340
Gly	0.0728	3.750
Thr	0.0660	3.402
Ala	0.0685	3.532
Arg	0.1379	7.109
Tyr	0.0539	2.779
Val	0.0822	4.236
Met	0.0231	1.192
Phe	0.0917	4.725
Ile	0.0826	4.256
Leu	0.1355	6.987
Lys	0.1207	6.223
Pro	0.0780	4.021
	w_{tot} = 1.6717	ratio % tot = 86.172

7. Edman Degradation Analysis

For amino acid sequence determination, fraction A1 was submitted to automatic Edman degradation (G1000A Hewlett Packard) according to manufacturer methods.

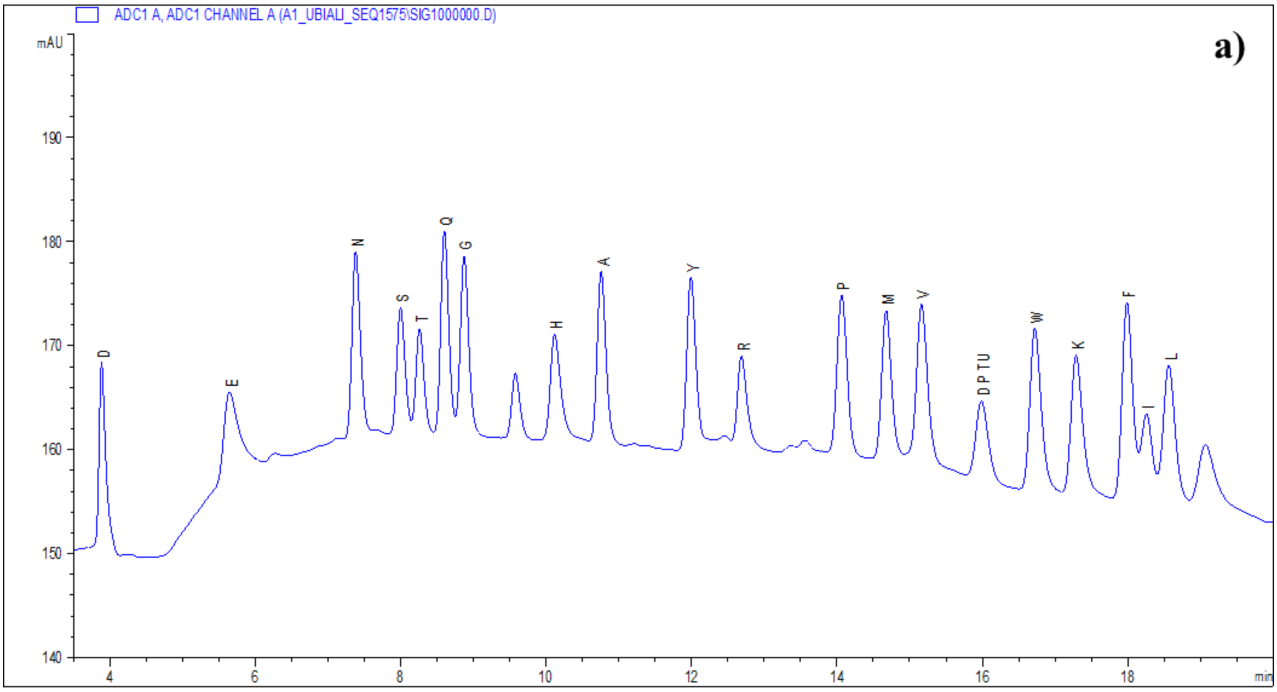

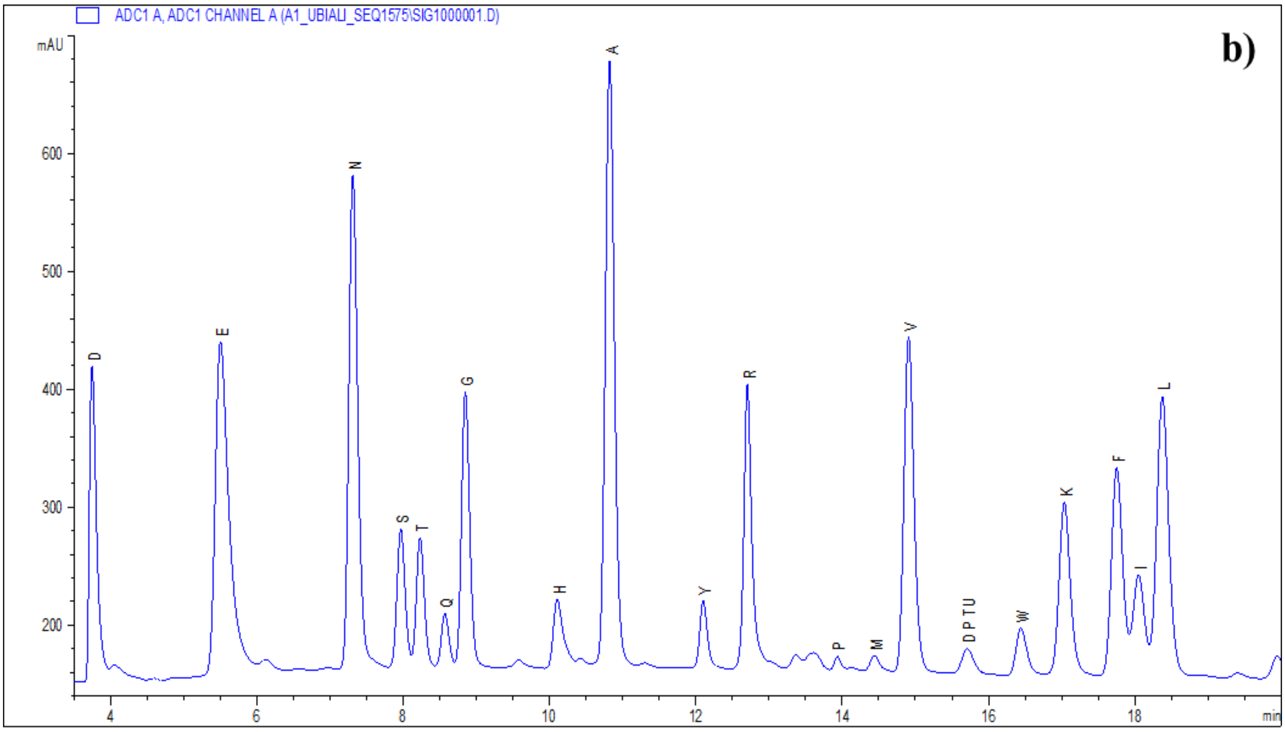
ADC1 A, ADC1 CHANNEL A (A1_UBIALI_SEQ1575\SIG1000001.D)
b)
mAU
600
500
400
300
200
D
E
N
S
T
Q
G
H
A
Y
R
P
M
V
D P T U
W
K
F
I
L
4
6
8
10
12
14
16
18
min

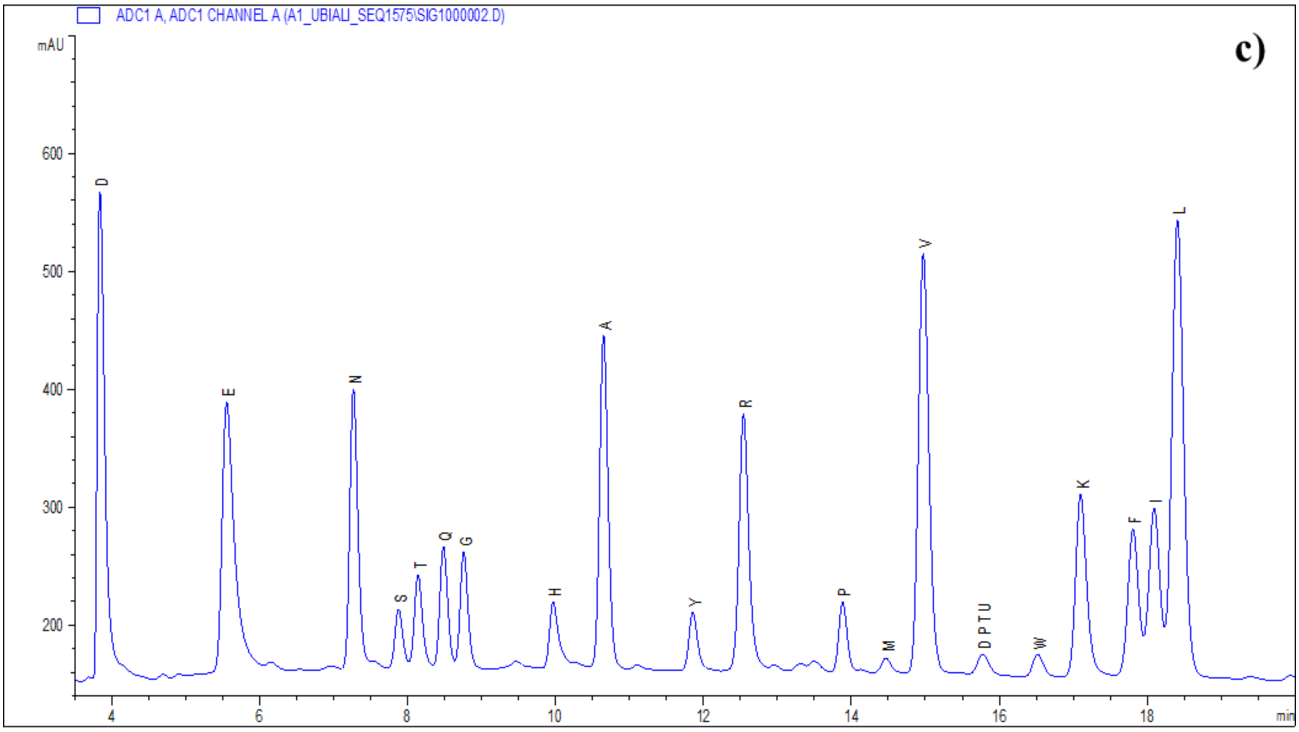
ADC1 A, ADC1 CHANNEL A (A1_UBIALI_SEQ1575\SIG1000002.D)
c)
mAU
600
500
400
300
200
D
E
N
S
T
Q
G
H
A
Y
R
P
M
V
D P T U
W
K
F
I
L
4
6
8
10
12
14
16
18
min

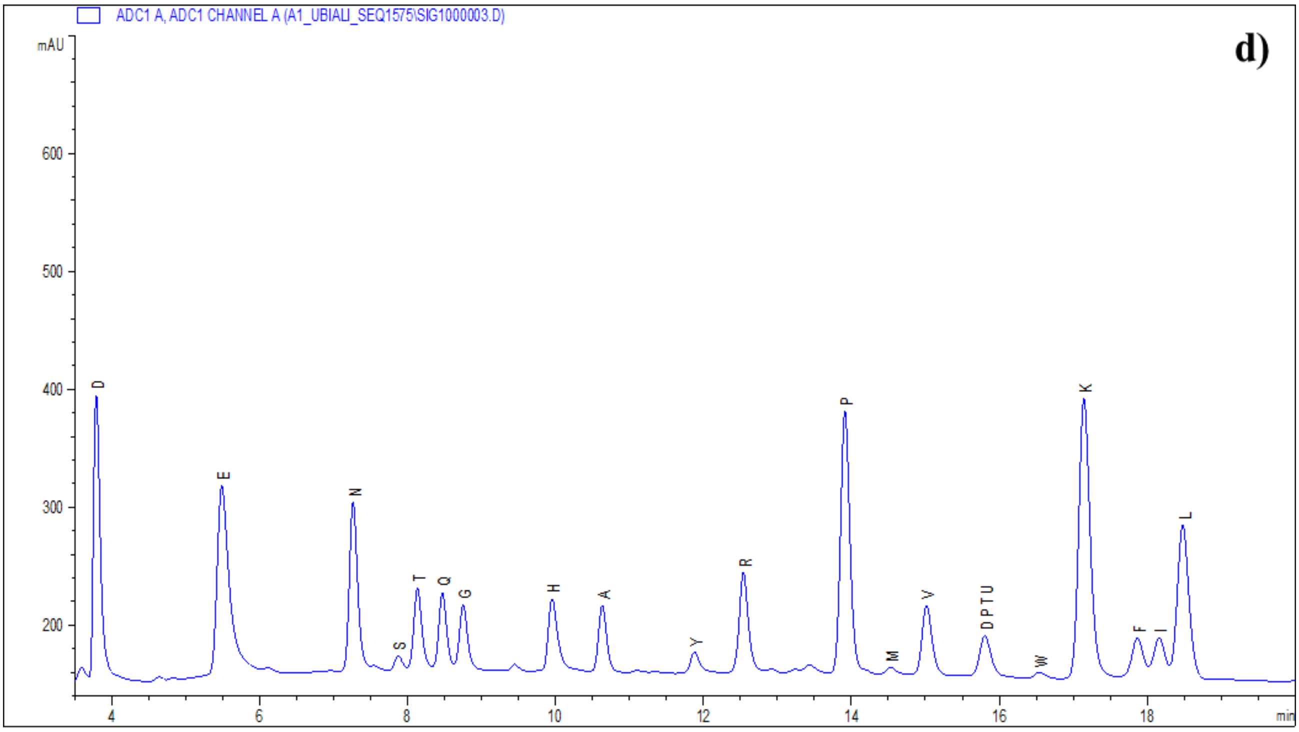
ADC1 A, ADC1 CHANNEL A (A1_UBIALI_SEQ1575\SIG1000003.D)
d)
mAU
600
500
400
300
200
4
6
8
10
12
14
16
18
min
D
E
N
S
T
Q
G
H
A
Y
R
P
M
V
DPTU
W
K
F
I
L

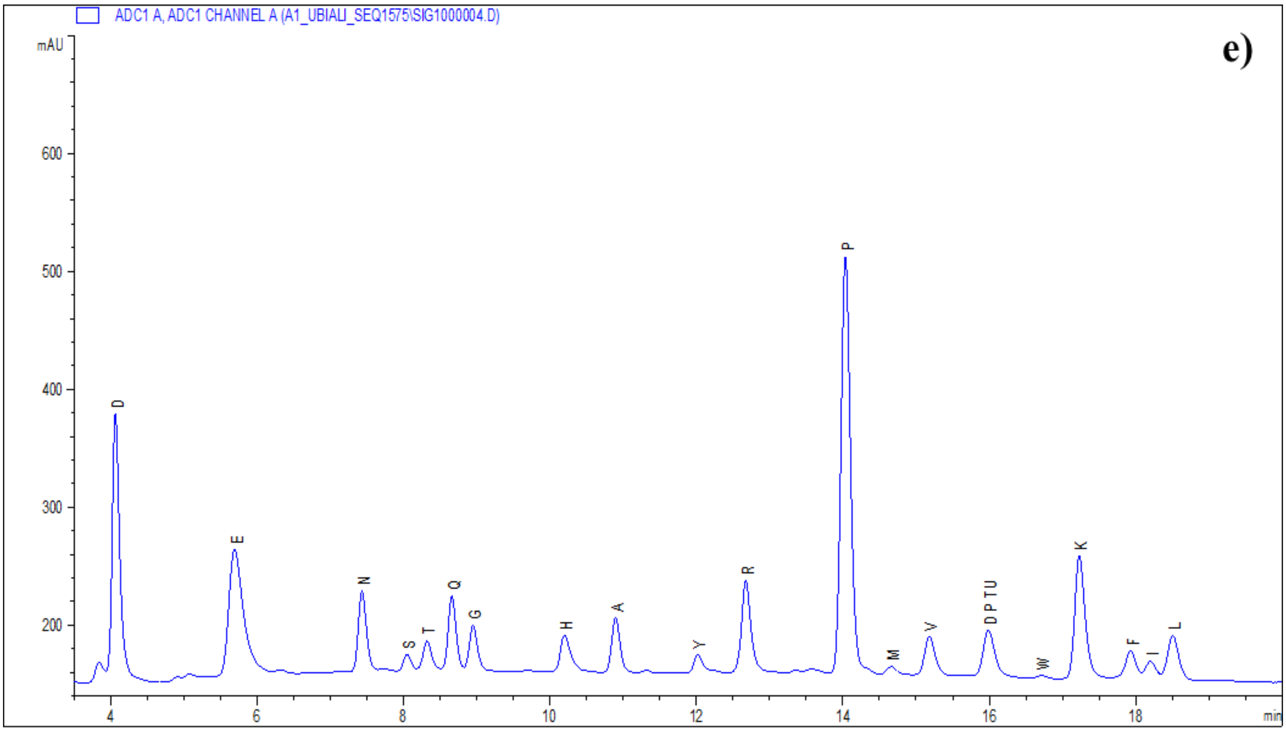
ADC1 A, ADC1 CHANNEL A (A1_UBIALI_SEQ1575\SIG1000004.D)
e)
mAU
600
500
400
300
200
4
6
8
10
12
14
16
18
min
D
E
N
S
T
Q
G
H
A
Y
R
P
M
V
DPTU
W
K
F
I
L

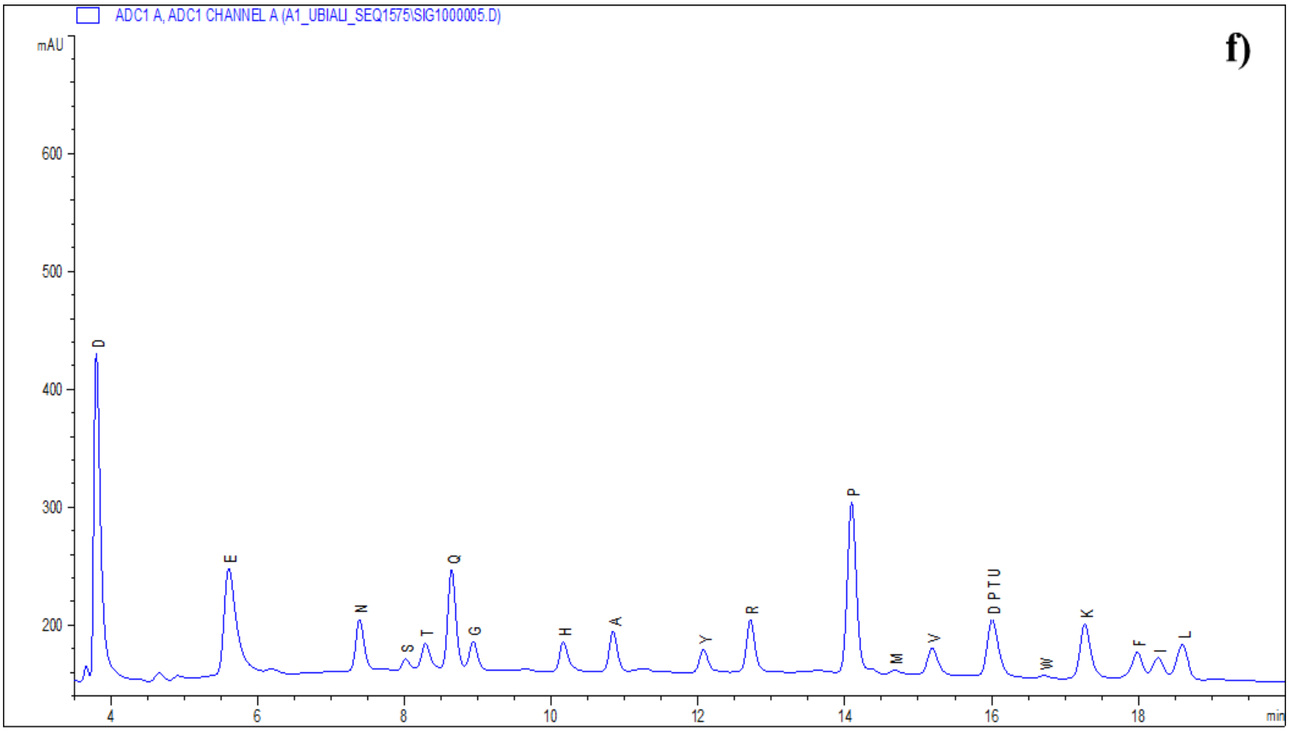
ADC1 A, ADC1 CHANNEL A (A1_UBIALI_SEQ1575\SIG1000005.D)
f)
mAU
600
500
400
300
200
D
E
N
S
T
Q
G
H
A
Y
R
P
M
V
DPTU
W
K
F
I
L
4
6
8
10
12
14
16
18
min

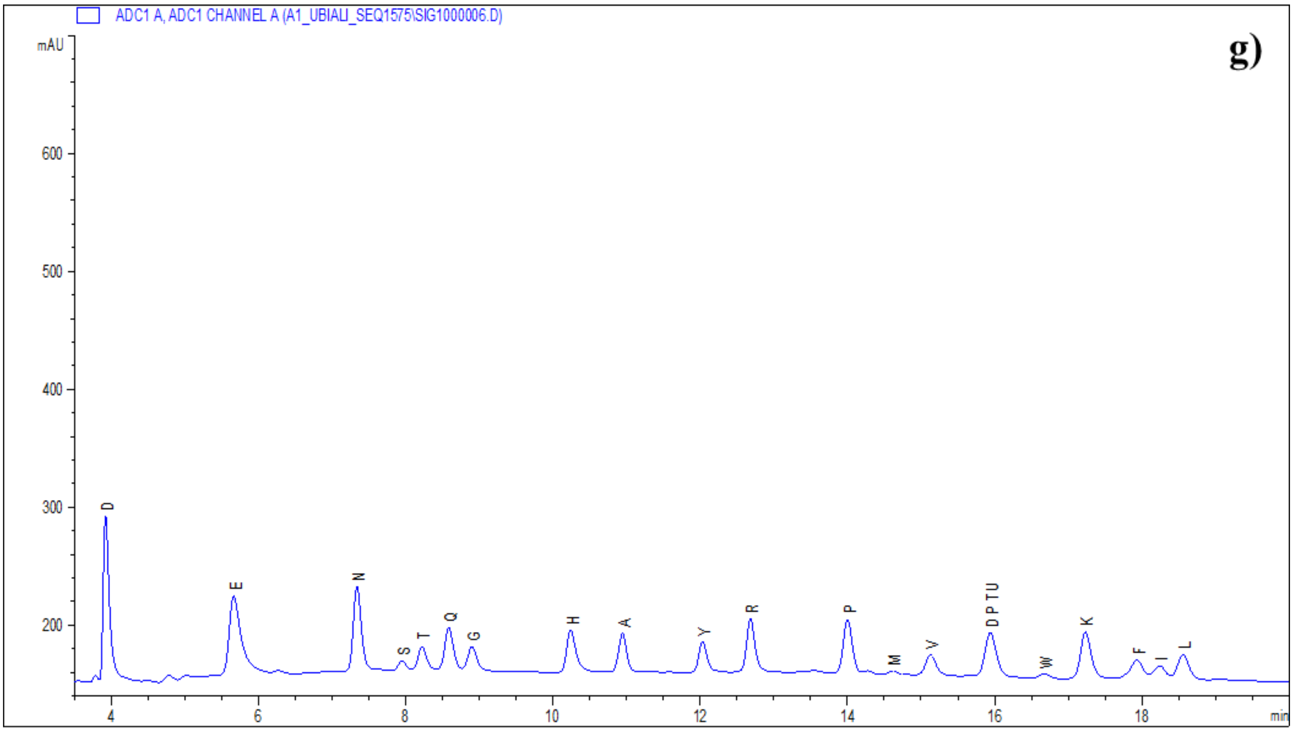
ADC1 A, ADC1 CHANNEL A (A1_UBIALI_SEQ1575\SIG1000006.D)
g)
mAU
600
500
400
300
200
D
E
N
S
T
Q
G
H
A
Y
R
P
M
V
DPTU
W
K
F
I
L
4
6
8
10
12
14
16
18
min

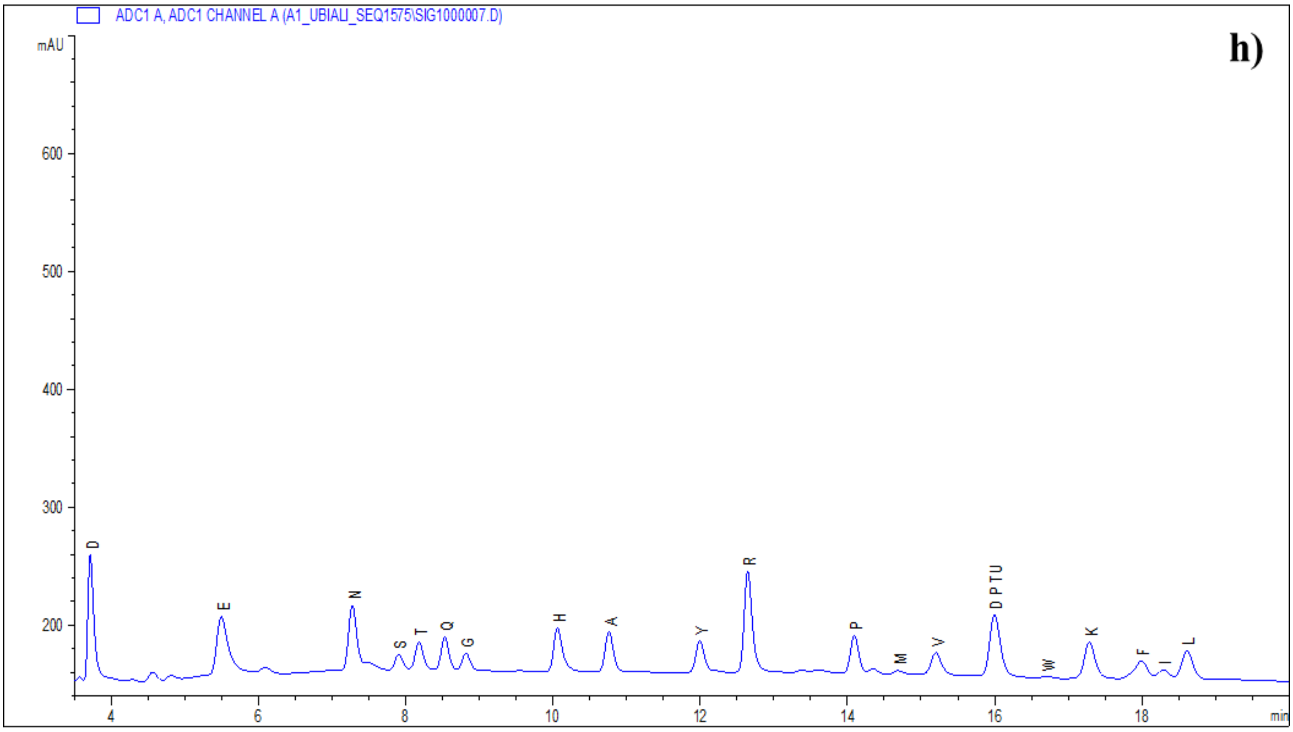

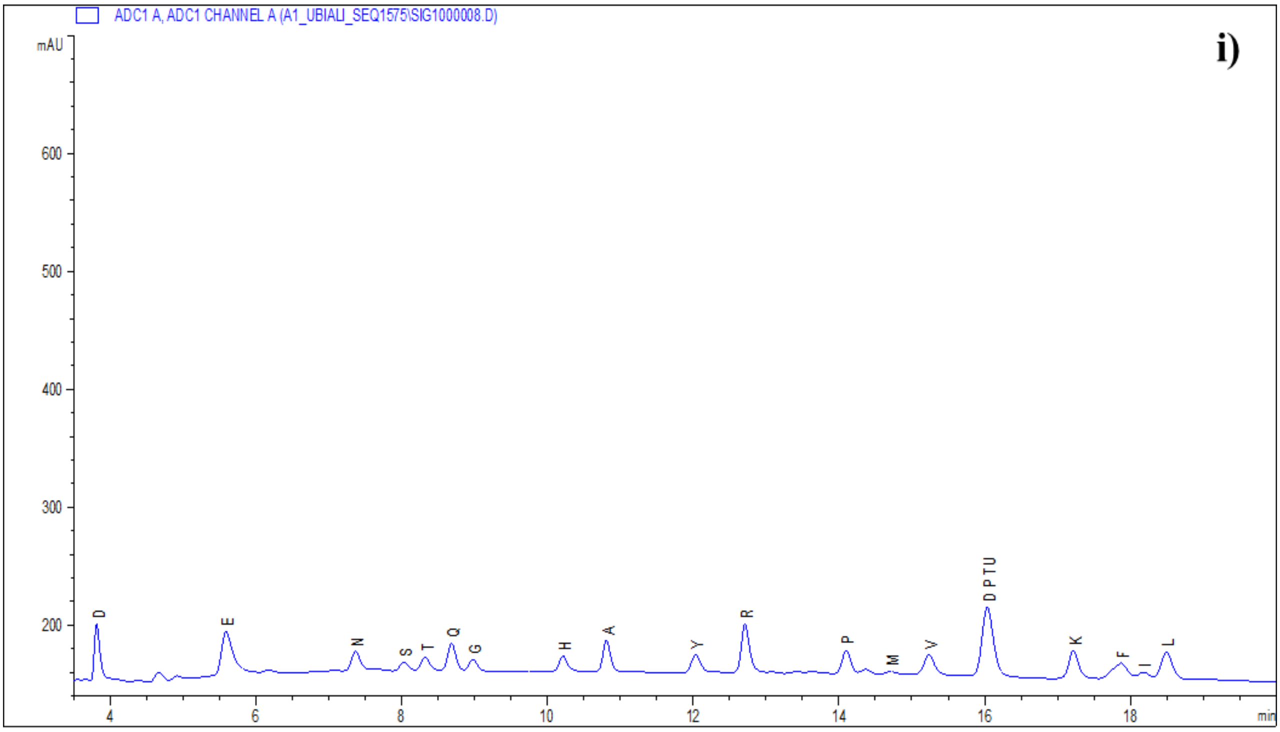

Figure S10. HPLC chromatograms of (**a**) PTH-Std and (**b**), (**c**), (**d**), (**e**), (**f**), (**g**), (**h**), (**i**) 8 cycles of Edman degradation.

8. Peptides Loading on Resin Support for Solid Phase Synthesis

In a plastic syringe equipped with a filter, 2-chlorotrityl chloride resin (500 mg, loading: 1.6 mmol/g) was suspended in anhydrous dichloromethane (DCM) (3 mL) and stirred for 1 h. Depending on the peptide, Fmoc-Arg(Pbf)-OH (390 mg, 0.6 mmol), Fmoc-Asp(OtBu)-OH (247 mg, 0.6 mmol), or Fmoc-Pro-OH (253 mg, 0.75 mmol) and *N*,*N*-diisopropylethylamine (DIPEA) (0.53 mL, 3 mmol) were added and the mixture was stirred for 1.5 hours. In the case of proline, a second loading was performed in the same condition to ensure the loading of the first amino acid to the resin. Solvent was then removed and resins were washed using a DCM/MeOH/DIPEA (17:2:1) mixture (3 × 3 mL), DCM (3 × 3 mL), *N*,*N*-dimethylformamide (DMF) (2 × 3 mL) and DCM again (2 × 3 mL) and finally dried under vacuum condition. 629 mg, 640 mg and 727 mg of dry resins were obtained, corresponding to a Fmoc-Arg(Pbf)-OH, Fmoc-Asp(OtBu)-OH and Fmoc-Pro-OH loading of about 0.88, 0.68 and 1.34 mmol/g, respectively.

9. Spectroscopic Characterization of Synthesized and In Vitro Digested Peptides

Synthesized peptides were submitted to electrospray ionization mass spectroscopy (ESI-MS), ^{1}H NMR, ^{13}C NMR and high-resolution mass spectroscopy (HR-MS) analysis.

9.1. ALKPDNR ESI-MS (Positive Detection)

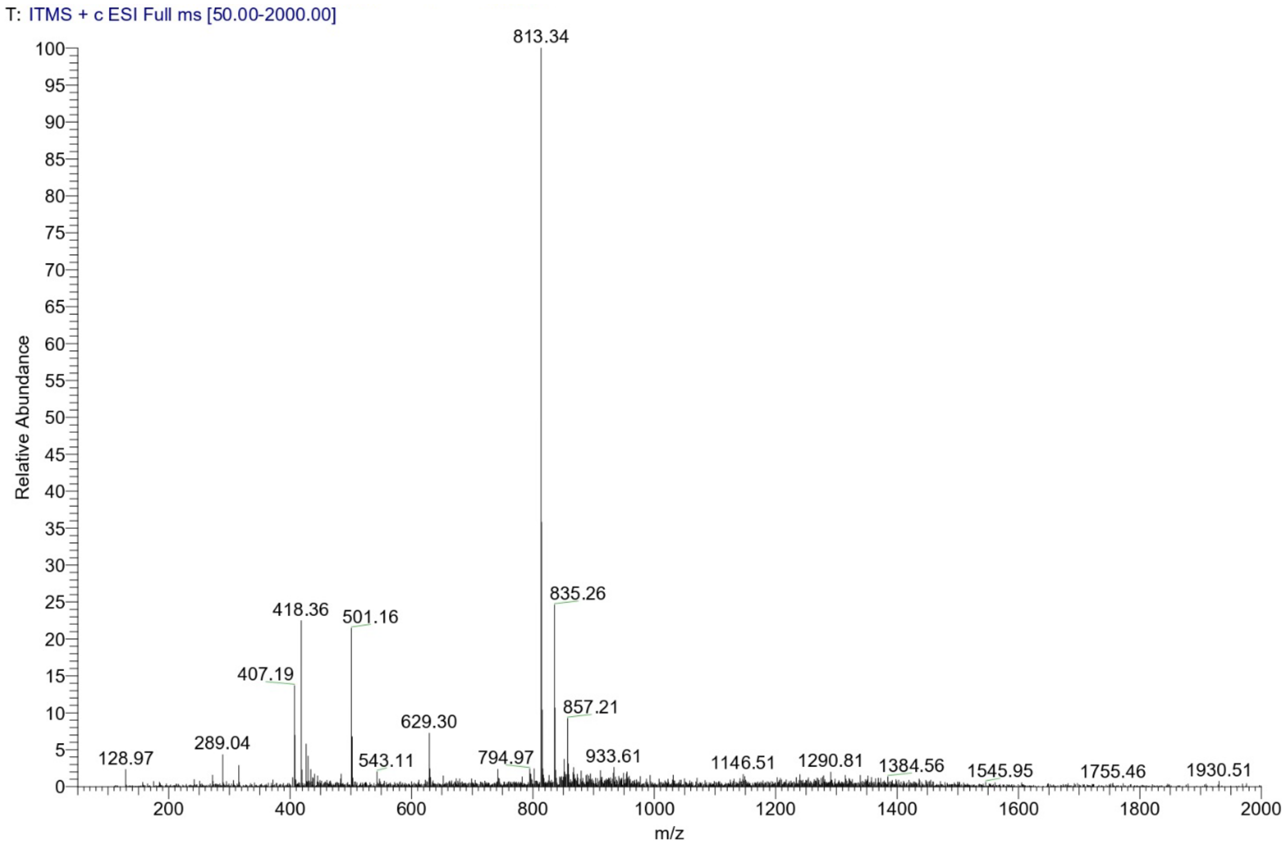

Figure S11. Mass spectra of ALKPDNR carried out in positive ion mode.

9.2. VVPD ESI-MS (Positive Detection)

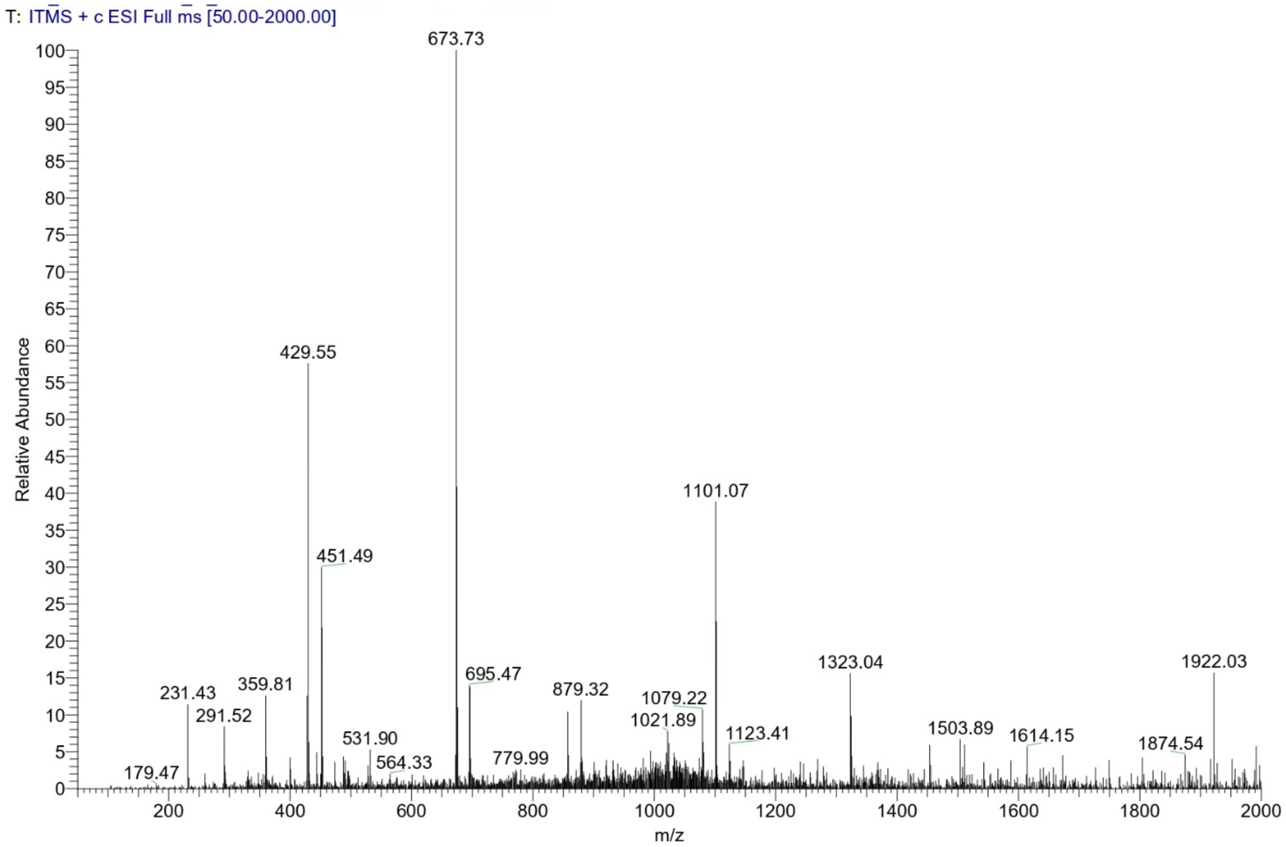

Figure S12. Mass spectra of VVPD carried out in positive ion mode.

9.3. NDRP ESI-MS (Positive Detection)

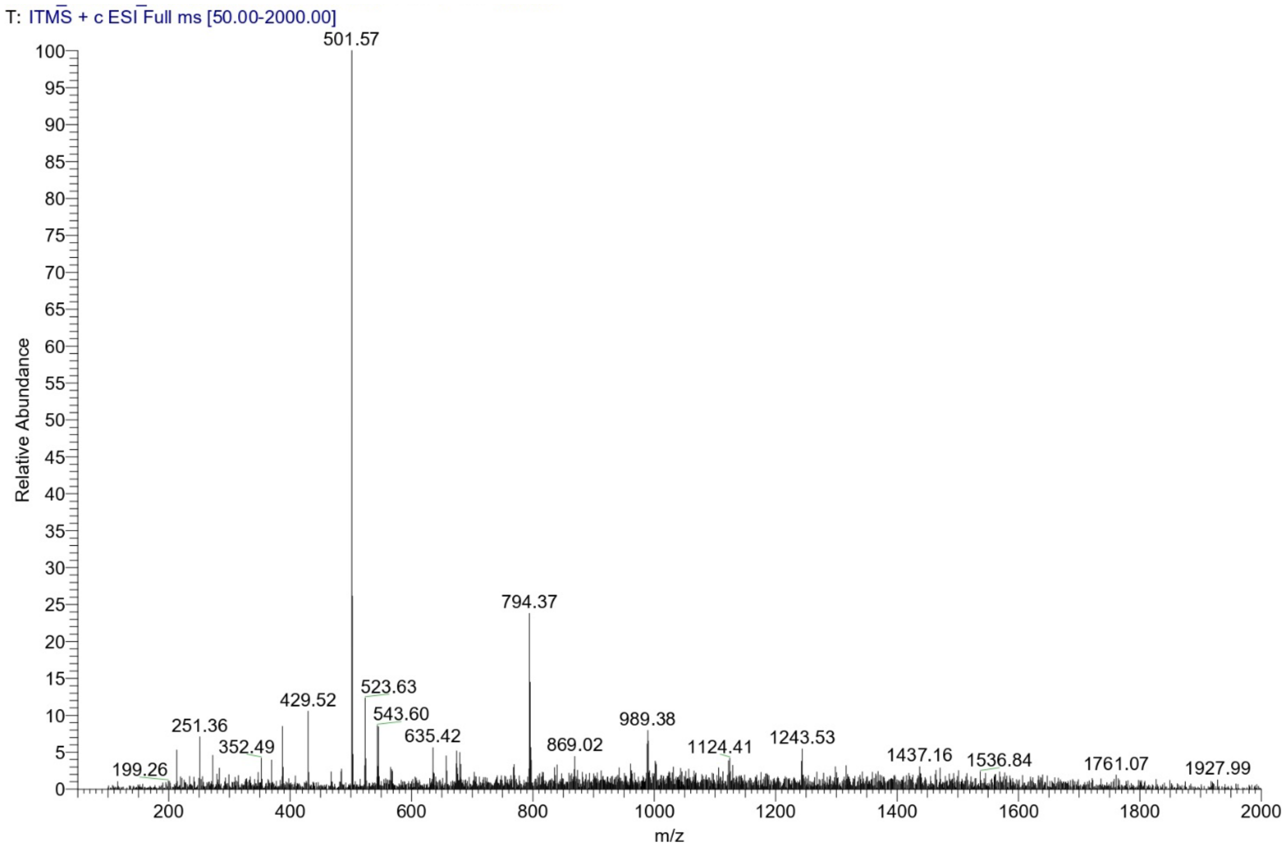

Figure S13. Mass spectra of NDRP carried out in positive ion mode.

9.4. NDTP ESI-MS (Positive Detection)

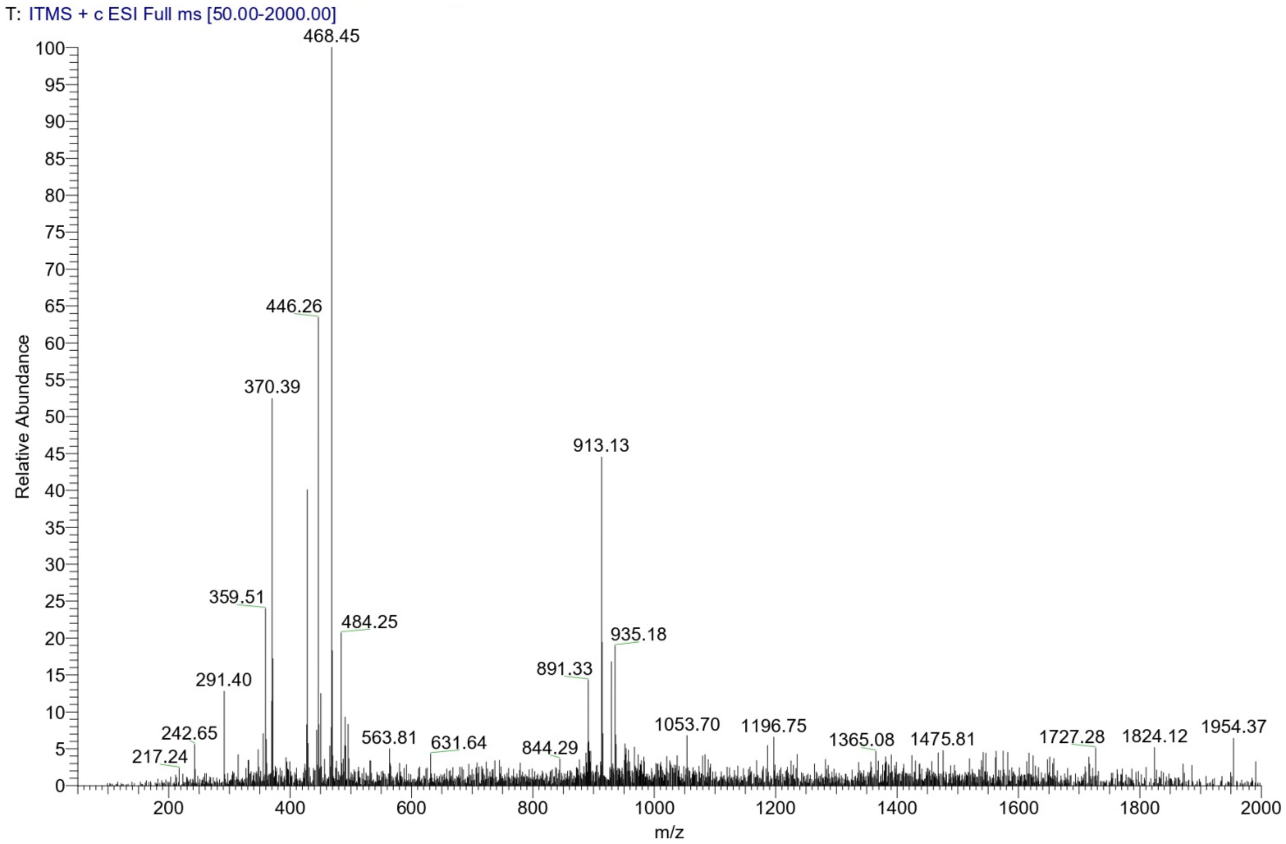

Figure S14. Mass spectra of NDTP carried out in positive ion mode.

9.5. In Vitro Digested NDRP ESI-MS (Positive Detection)

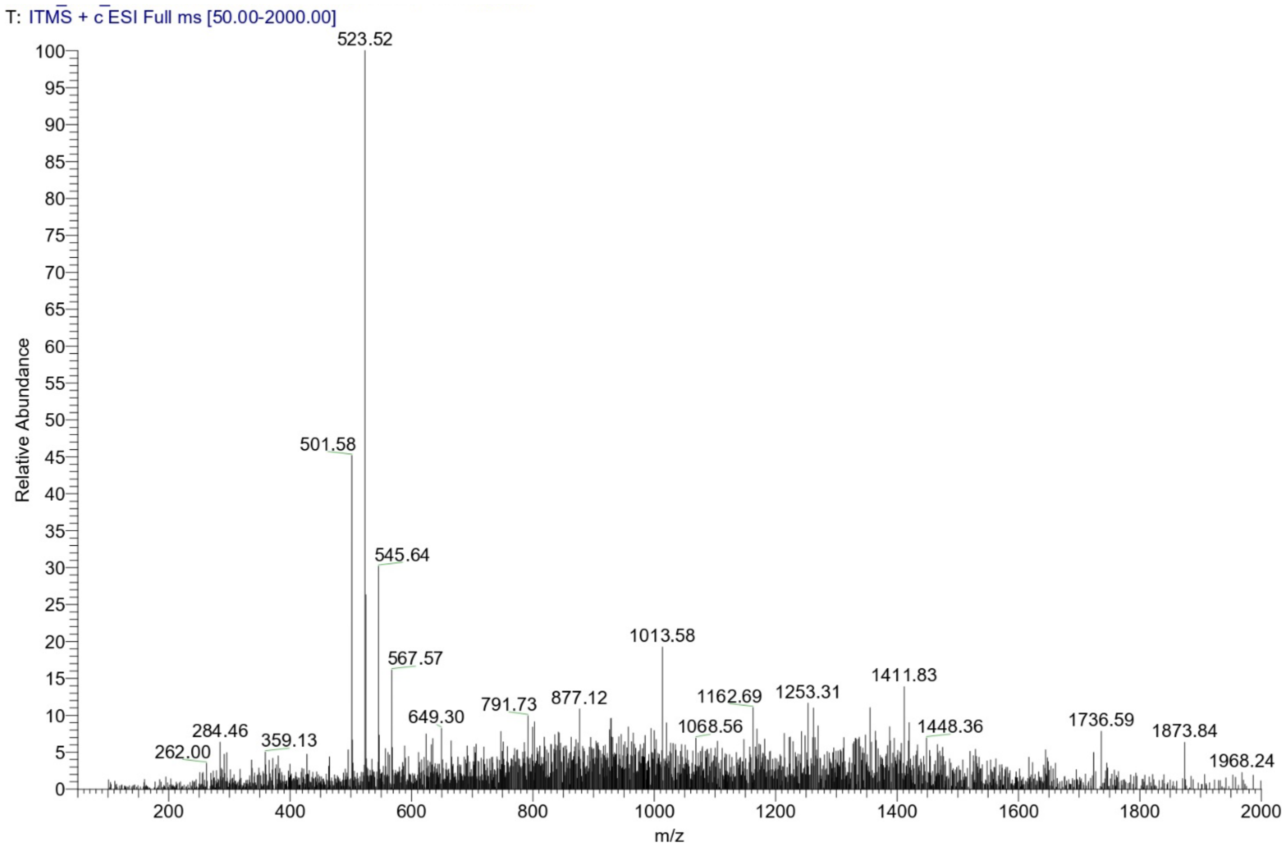

Figure S15. Mass spectra of in vitro digested NDRP carried out in positive ion mode.

9.6. ¹H NMR of NDRP

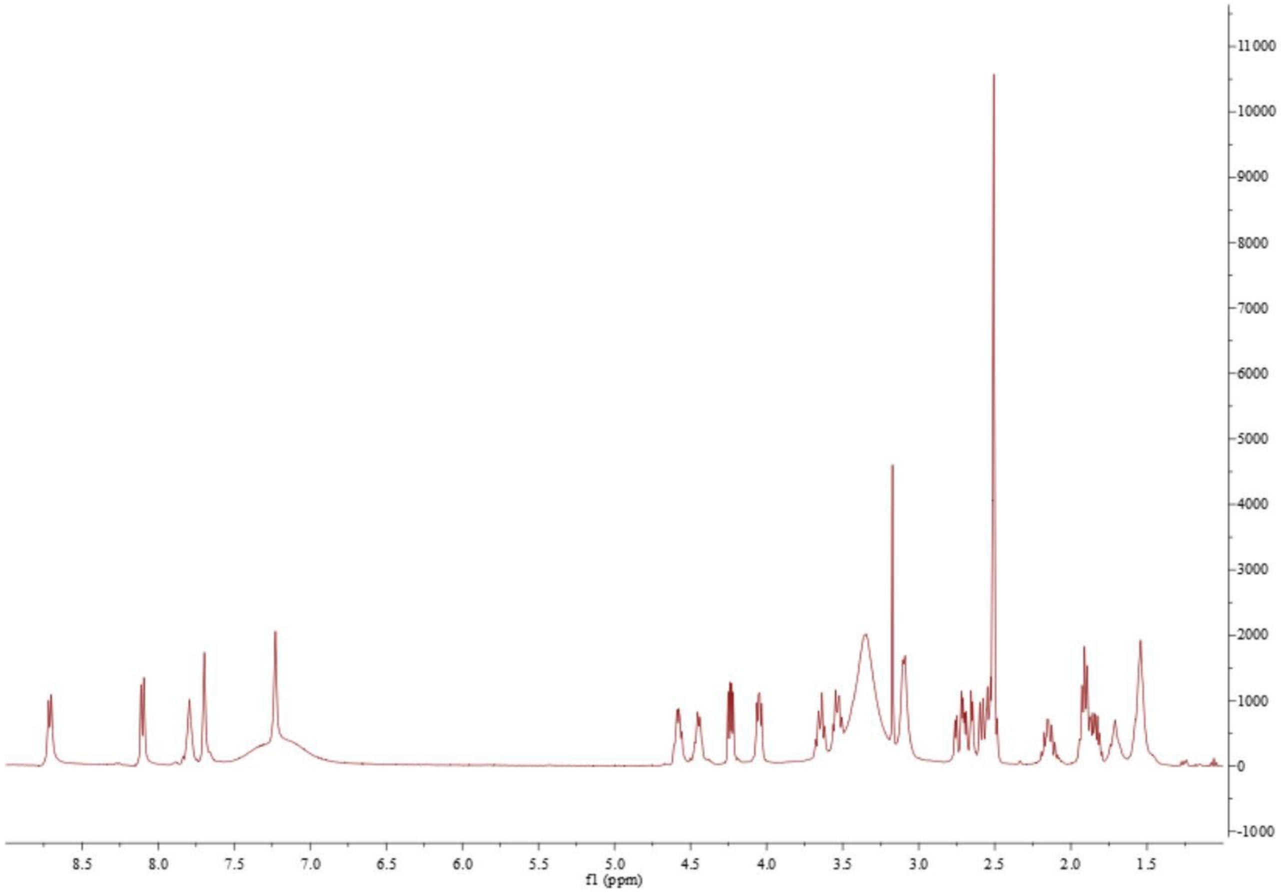

Figure S16. ¹H NMR (400 MHz) spectra of NDRP recorded in DMSO-d_6.

9.7. ^{13}C NMR of NDRP

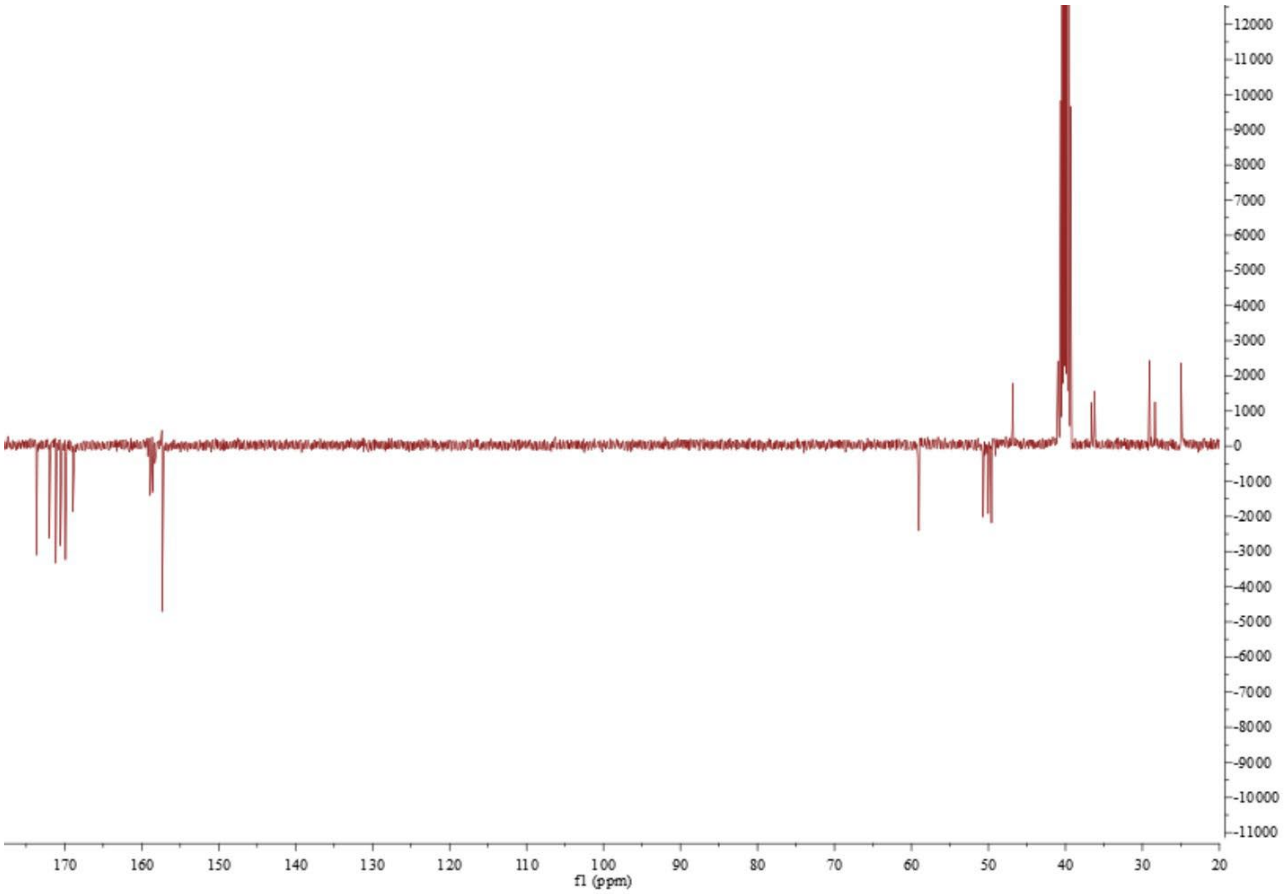

Figure S17. ^{13}C NMR (101 MHz) spectra of NDRP recorded in DMSO-d_6.

9.8. NDRP HR-MS (Positive Detection)

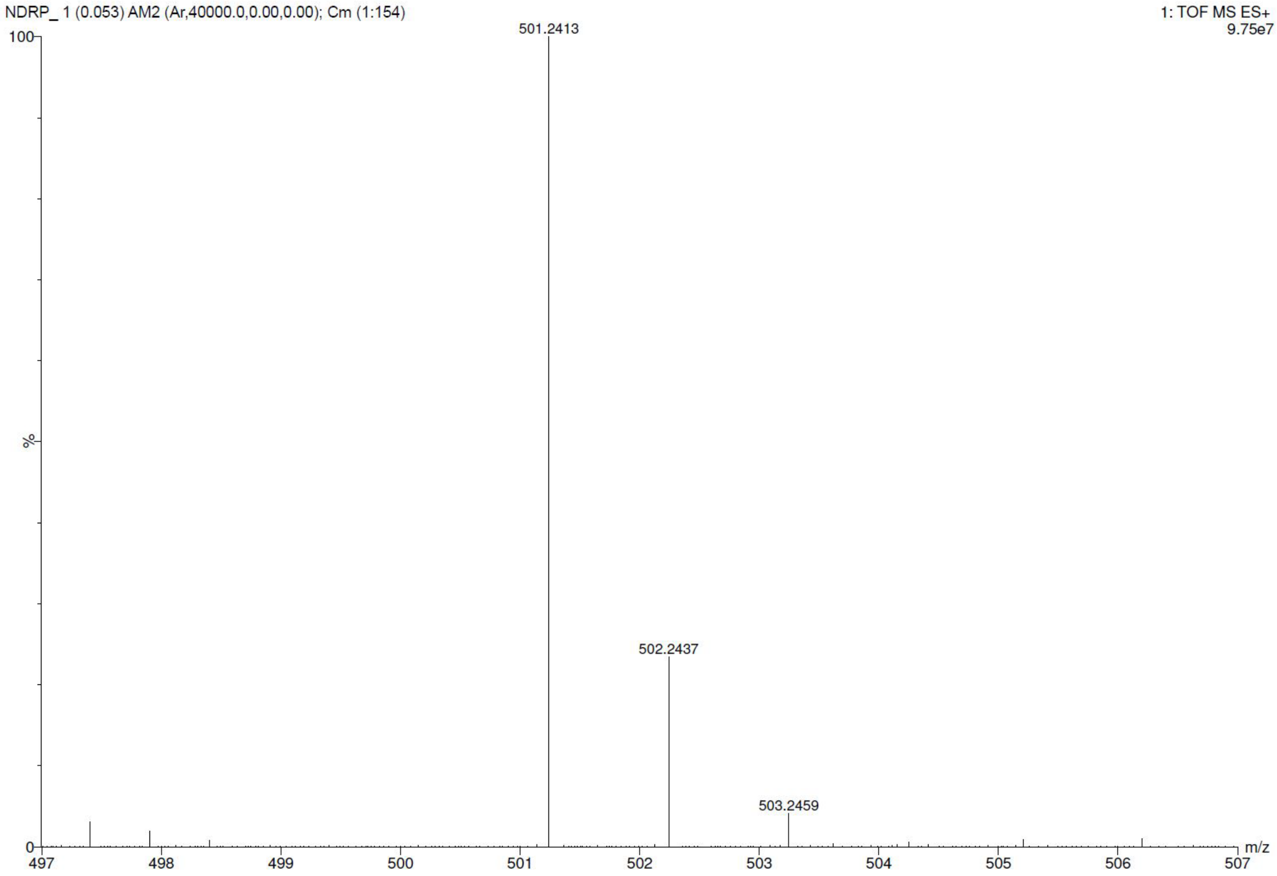

Figure S18. High-resolution ESI mass spectra (positive ion mode) of NDRP.
